# Metabolic and hormonal effects of melatonin and/or magnesium supplementation in women with polycystic ovary syndrome: a randomized, double-blind, placebo-controlled trial

**DOI:** 10.1186/s12986-021-00586-9

**Published:** 2021-06-06

**Authors:** Mohammad Alizadeh, Majid Karandish, Mohammad Asghari Jafarabadi, Lida Heidari, Roshan Nikbakht, Hossein Babaahmadi Rezaei, Reihaneh Mousavi

**Affiliations:** 1 Department of Clinical Nutrition, Faculty of Nutrition and Food Science, Tabriz University of Medical Science, Tabriz, Iran; 2 Nutrition and Metabolic Disease Research Center, Clinical Science Research Institute, Ahvaz Jundishapur University of Medical Science, Ahvaz, Iran; 3 Department of Statistics and Epidemiology, School of Medicine, Zanjan University of Medical Sciences, Zanjan, Iran; 4 Road Traffic Injury Research Center, Tabriz University of Medical Science, Tabriz, Iran; 5 Nahal Infertility Center, Tabriz, Iran; 6 Fertility Infertility and Perinatology Research Center, Ahvaz Jundishapur University of Medical Science, Ahvaz, Iran; 7 Hyperlipidemia Research Center, Department of Clinical Biochemistry, Faculty of Medicine, Ahvaz Jundishapur University of Medical Science, Ahvaz, Iran; 8 Department of Nutrition, School of Allied Medical Sciences, Ahvaz Jundishapur University of Medical Sciences, Ahvaz, Iran; 9 Student Research Committee, Ahvaz Jundishapur University of Medical Sciences, Ahvaz, Iran

**Keywords:** Polycystic ovary syndrome, Melatonin, Magnesium, Metabolic profile

## Abstract

**Background:**

Polycystic ovary syndrome (PCOS) is one of the most common endocrine disorders among women of reproductive age. This study was designed to investigate the effects of melatonin and/or magnesium supplementation on metabolic profile and levels of sex hormones in PCOS women.

**Methods:**

In an 8-week randomized double-blind placebo-controlled trial, 84 subjects with PCOS aged 18–40 years were randomly assigned based on the random block procedure to take magnesium, melatonin, magnesium plus melatonin, and placebo. Fasting blood samples were obtained at the beginning and end of the study.

**Results:**

After intervention, the mean Pittsburg Sleep Quality Index score decreased significantly in both co-supplementation and melatonin groups (*P* < 0.001). Magnesium supplementation in combination with melatonin resulted in a significant greater decrease in testosterone concentrations compared with the placebo (*P* < 0.05). Co-supplementation of magnesium-melatonin had significantly reduced serum insulin levels (geometric means difference: − 1.11 (mIU/mL) (percent change: − 15.99)), homeostasis model of assessment-insulin resistance (HOMA-IR) (− 0.28 (− 18.66)), serum cholesterol (mean difference: − 16.08 (mg/dl) [95% CI − 24.24, − 7.92]), low-density lipoprotein cholesterol (LDL-C) − 18.96 (mg/dl) [− 28.73, − 9.20]) and testosterone levels (− 0.09 (ng/ml) (− 25.00)), as compared to the baseline values (*P* < 0.05). An increase in serum high-density lipoprotein cholesterol (HDL-C) levels was also observed following the administration of the melatonin alone (2.76 (mg/dl) [0.57, 4.95]) or in combination with magnesium (2.19 (mg/dl) [0.61, 3.77]) (*P* < 0.05).

**Conclusions:**

Co-supplementation with magnesium and melatonin had beneficial effects on sleep quality and total testosterone. Additionally, melatonin supplementation alone was found to be associated with a significant reduction in PSQI score. Moreover, combined melatonin and magnesium supplementation was more effective in improving serum levels of cholesterol, LDL-C, HDL-C and insulin, and HOMA-IR.

*Trial registration*: Iranian Registry of Clinical Trial. http://www.irct.ir: IRCT20191130045556N1, January 2020.

## Background

Polycystic ovary syndrome (PCOS) is one of the most common endocrine disorders affecting about 7% to 10% of women of reproductive age and is a leading cause of infertility [1, 2]. The clinical expression of the syndrome is characterized by the manifestation of oligo/anovulation, clinical or biochemical hyperandrogenism and/or polycystic ovaries [3]. PCOS is a heterogeneous gynecological syndrome, associated with a wide range of endocrine and metabolic abnormalities, including hyperinsulinemia, hyperglycemia, glucose intolerance, dyslipidemia, and obesity, which are regarded as the components of metabolic syndrome (MetS) [4]. Insulin resistance (IR) with compensatory hyperinsulinemia plays a major role in the development of PCOS. Insulin excess stimulates androgen synthesis in the ovary and the adrenals [5–7]; besides, it can inhibit sex hormone-binding globulin (SHBG) synthesis in the liver [5] and increase the levels of free testosterone (T) [8, 9]. On the other hand, Adipokines such as leptin, as a product of the obesity gene, plays a crucial role in body weight homeostasis through possible neuroendocrine pathways [10–12] and has an impact on gonadal function and reproduction [13, 14]. It may contribute to the development of type 2 diabetes mellitus, and IR; therefore, leptin may be involved in the pathogenesis of PCOS [15]. Lifestyle modifications are first-line treatment for PCOS, and small lifestyle changes (diet, exercise, and behavior) can improve metabolic dysfunction, ovulation, fertility, and mood [16, 17]. Nutritional supplements had received a great deal of attention in the management of PCOS [18].

Melatonin is the main hormone that is mainly secreted by the pineal gland to regulate circadian rhythms, reproduction, and the sleep cycle [19, 20]. High levels of melatonin in the follicular fluid are essential for folliculogenesis, ovulation, and oocyte quality, whereas reduced follicular melatonin concentrations may be responsible for anovulation and poor oocyte quality in PCOS [21]. It had been suggested that melatonin has an antigonadal effect through the direct reduction of testosterone production [22]. Also, beneficial effects of melatonin on the components of MetS, including hyperglycemia, dyslipidemia, and insulin resistance, have been shown in both animal and human studies [23, 24]. In a randomized controlled trial, Melatonin administration for 12 weeks in PCOS patients had beneficial effects on mental health parameters, insulin levels, homeostasis model of assessment-insulin resistance (HOMA-IR), the quantitative insulin sensitivity check index (QUICKI), total- and low-density lipoprotein cholesterol (LDL-C) levels [25].

On the other hand, the function of minerals, including magnesium (Mg), in the pathogenesis of PCOS due to its contribution to insulin sensitivity has been examined [26]. It has been shown that hypomagnesemia increase PCOS risk by up to 19 times [27]. There is evidence that Mg increases insulin sensitivity through its influence on tyrosine-kinase activity, and its deficiency is associated with IR [26]. Beneficial effects on parameters of insulin metabolism and serum triglycerides, and total cholesterol in PCOS women have been shown in 12-week magnesium and vitamin E co-supplementation [28]. Also, an 8-week Magnesium supplementation resulted in reduced body mass index (BMI) and testosterone levels as well as increased serum dehydroepiandrosterone (DHEA) and luteinizing hormone (LH) levels in women with PCOS, but it did not affect serum lipid profiles and glycemic indicators, follicle-stimulating hormone, 17OH progesterone, SHBG, and free androgen index (FAI) levels [29]. Despite the relationships between Mg levels and PCOS status, few studies have evaluated magnesium supplementation in the management of PCOS.

We hypothesized that melatonin and magnesium co-supplementation might help improve metabolic profiles and clinical symptoms of PCOS and may work better than a single supplementation alone. The present study was carried out to investigate the effect of melatonin and magnesium supplementation, separately and together, on metabolic profiles and levels of sex hormones in women with PCOS.

## Methods

### Trial design

This randomized double-blind placebo-controlled clinical trial was performed in Alzahra and 29 Bahman hospitals of Tabriz, Iran, from April 2020 through December 2020. The protocol of this study was approved by the Medical Ethics Committee of Ahvaz Jundishapur University of Medical Sciences which is in accordance with the Declaration of Helsinki (approval number IR.AJUMS.REC.1398.637). Also, this trial has been registered in the Iranian Registry of Clinical Trials (IRCT) with the number of IRCT20191130045556N1. Additionally, written informed consent was obtained from all participants.

### Participants

Diagnosis of PCOS was performed according to the Rotterdam criteria [30]. The participants were females with age ranges between 18 and 40 and body mass index (BMI) ≤ 35. The exclusion criteria were as follows: pregnancy, lactation, smoking, alcohol consumption, endocrine disorders, in particular any type of adrenal diseases, diabetes, hyperprolactinemia, receiving drugs affecting plasma androgen levels, lipid profile, or inflammatory factors during the last 3 months, weight loss of more than 5% in the last six months, supplementation with melatonin, magnesium, antioxidant and/or anti-inflammatory agents within the last three months and those with sleeping disorders and night shift working.

### Interventions

Participants were randomly assigned into four groups. Subjects in four groups were receiving: two melatonin tablets (each, 3 mg) plus a 250 mg magnesium oxide tablet (group one); two melatonin tablets (each, 3 mg) plus a magnesium placebo (group two); a 250 mg magnesium oxide tablet plus two melatonin placebos (group three), and two melatonin placebos plus a magnesium placebo (group four) for eight consecutive weeks. Melatonin and magnesium supplements were manufactured by Nature Made Pharmaceutical Company (California, USA) and Jalinous Pharmaceutical Company (Tehran, Iran), respectively. Placebos were provided by Pharmacy faculty, Tabriz University of Medical Science, Tabriz, Iran. The appearance of placebos, including color, shape, size, and packaging, was identical to that of melatonin and magnesium supplements. The participants were asked to take their melatonin tablets at night before sleeping and magnesium tablets in the evening for eight consecutive weeks.

At the onset of the study, the patients were asked to maintain their usual dietary pattern and physical activity level during the study. Additionally, patients were requested not to receive any antioxidant and/or anti-inflammatory, and other medications that could affect their reproductive physiology during the intervention. Compliance with the intake of supplements or placebos was checked by asking participants to bring the medication containers. Further, all participants were contacted two times per week by a dietitian. The participants were allowed to discontinue the trial if they were unwilling to complete the trial or if they experienced any adverse effects during the intervention. Consuming more than 90% of the supplements was considered compliant.

### Dietary intakes and physical activity assessment

Dietary intakes were estimated from three 24 h dietary recalls (one weekend day and two weekdays). To obtain nutrient intakes of participants based on these three dietary recalls, we used Nutritionist IV software (First Databank, San Bruno, CA). Physical activity levels were determined by the short form of the International Physical Activity Questionnaire Short Form (IPAQ-S) at baseline and after eight weeks of intervention. Further, total physical activity is expressed as metabolic equivalents (METs) minutes/week [31].

### Sleep quality assessment

Sleep quality was determined using the validated Iranian version of the Pittsburg Sleep Quality Index (PSQI) [32].

### Assessment of anthropometric variables

At baseline and end-of-trial, all subjects underwent standard anthropometric measurements: height was measured using a non-stretched tape measure (Seca, Hamburg, Germany) to the nearest 0.1 cm. Body weight was measured in a minimal clothing state and no shoes using a digital scale (Seca, Hamburg, Germany) to the nearest 0.1 kg. BMI was calculated as weight in kg divided by height in meters squared. Waist circumference (WC) (the widest area between the lower rib and the superior iliac crest) was also measured to the nearest 0.1 cm.

### Laboratory analysis

Blood samples were collected after 12 h overnight fasting before and after the intervention, and serum was obtained by centrifugation at 3000 RPM for 10 min; the serum samples were frozen and stored at − 80 °C until biochemical analyses. Fasting blood sugar (FBS), TG, total cholesterol (TC), high-density lipoprotein cholesterol (HDL-C), and magnesium were measured using a colorimetric method (Parsazmoun, Tehran, Iran). The concentration of LDL-C was calculated using the Friedewald formula [33].

Serum insulin levels were measured by an enzyme-linked immune sorbent assay (ELISA) method using a laboratory kit (Monobind, USA). Serum testosterone and SHBG levels were measured by ELISA kit (DiaMetra, Italy). Free androgen index (FAI) was calculated as total testosterone divided by SHBG according to Azziz et al. Equation [34]. Serum leptin values were also measured using an ELISA kit (LDN, Nordhorn, Germany). The HOMA-IR and homeostatic model assessment β cell function (HOMA-B) were determined according to the suggested formulas [35].

### Sample size

The sample size for the present study was calculated by PASS 15 (version 15, PASS; NCSS, LLC, US) [36]. To determine the sample size, regression coefficient, and the confidence interval of the relationship between melatonin supplementation and serum insulin levels (reported by Shabani et al. [25]) were used (β: − 1.20 mIU/ml; 95% CI − 2.14, − 0.26). Considering a 95% confidence, a power of 95%, a two-tailed test, 1.2 unit change in slope, and a SD = 3 for insulin, the sample size was estimated to be 71 in total (18 per group). To consider the probable dropouts, 22 patients in the co-supplementation group, 21 patients in each melatonin and magnesium group and 20 patients in the placebo group were enrolled.

### Randomization

The patients were randomly assigned to intervention and placebo groups based on the random block procedure; for this, a third independent investigator who was not aware of the study clinical process created the randomization list assigning patients to the melatonin and magnesium co-supplementation, melatonin, magnesium, and placebo group. The random sequence was generated using random allocation software. Melatonin, magnesium, and placebo tablets were in the same form of a package. The study leader labeled these containers with patient numbers using the randomization list. All investigators and patients were blinded to the random assignments.

### Statistical analysis

The analyses were done based on an intention-to-treat approach. For doing this, missing values were treated based on the linear interpolation method. The distribution of data was examined using the Kolmogorov–Smirnov test. Numeric and categorical variables were presented as mean ± SD or geometric mean (min, max) where appropriate and frequency (percentage), respectively. For non-normally distributed variables, a log transformation was conducted before the analysis. Percent change of variables was calculated using the following equation: ((after–before)/before) × 100. A 1-way ANOVA test and a χ^2^ test were used to compare the four groups for baseline measures of the quantitative and qualitative data, respectively. Comparison of the four groups at the end of the study was completed using analysis of covariance followed by Sidak’s test after adjusting for baseline values and energy intake. The comparison of mean values was done within groups after the intervention using paired sample t-tests. Statistical analysis was performed using IBM SPSS Statistics (Version 22.0; IBM SPSS Statistics Inc. Armonk, USA). *P* < 0.05 was considered as statistically significant.

## Results

**Fig. 1:**
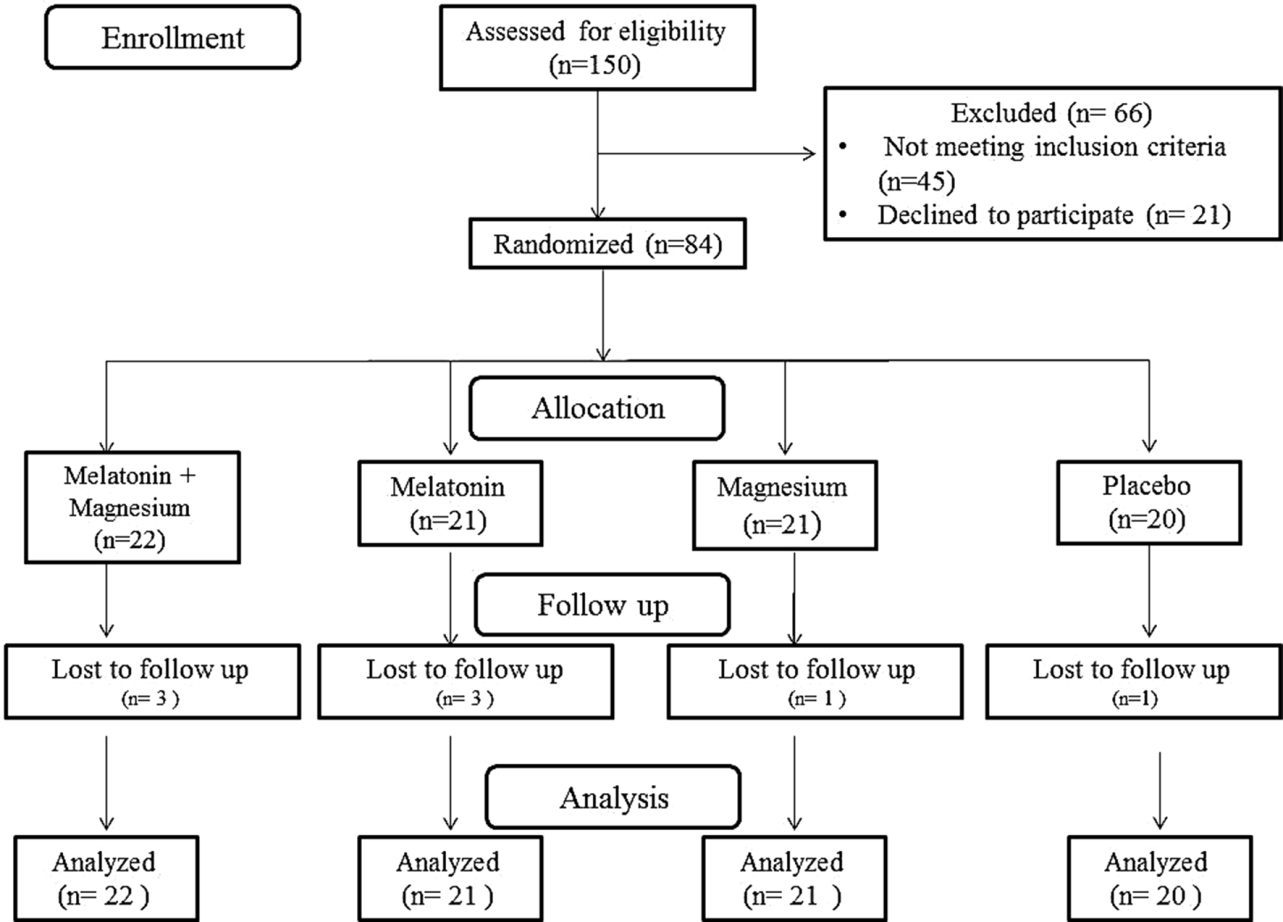
Summary of the patient flow diagram

In the current study, out of 84 participants who were selected for the intervention, seven subjects were excluded due to personal reasons [magnesium and melatonin co-supplementation (n = 2), melatonin (n = 3), placebo (n = 1), and magnesium (n = 1)]. Among participants in the magnesium plus melatonin supplements group, another woman did not complete the trial because of pregnancy. Finally, as the analyses were carried out according to the intention-to-treat approach, all 84 patients were included in the end analysis (Fig. 1). During the intervention, no adverse events or symptoms were reported by the patients.

There were no significant differences in terms of age, physical activity, family history of PCOS, irregular menstrual cycles, and height between different groups (Table 1). However, after eight weeks intervention, weight, BMI, and WC decreased significantly in the magnesium-melatonin co-supplementation (*P* < 0.05). Additionally, in comparison to baseline values, a significant reduction in WC was observed in the magnesium or melatonin supplementation alone (*P* < 0.05).

**Table 1:** General characteristics of study participants

Variables	The study groups				*P*
	Placebo (n = 20)	Melatonin + Mg (n = 22)	Melatonin (n = 21)	Mg (n = 21)	
	Mean ± SD	Mean ± SD	Mean ± SD	Mean ± SD	
Age (y)^a^	26.200 ± 5.72	28.22 ± 6.38	25.57 ± 4.99	25.57 ± 4.88	0.346
Family history of PCOS^c^	4 (20.00)	6 (27.30)	6 (28.60)	3 (14.30)	0.657
Irregular menstrual cycles^c^	20 (95.00)	22 (100.00)	20 (95.20)	19 (90.50)	0.541
Weight (kg)^a^					
Before	69.600 ± 11.39	76.61 ± 10.89	72.65 ± 11.21	71.23 ± 8.55	0.172
After	69.65 ± 11.26	76.02 ± 10.86	72.04 ± 11.28	70.61 ± 9.05	0.226
P*	0.888	0.018	0.061	0.127	
Height (cm)^a^	160.60 ± 6.46	160.63 ± 4.94	159.76 ± 5.19	159.57 ± 6.77	0.905
BMI (kg/m^2^)^a^					
Before	26.94 ± 3.83	29.64 ± 3.71	28.40 ± 3.86	27.99 ± 3.22	0.128
After	26.95 ± 3.72	29.41 ± 3.70	28.16 ± 3.83	27.74 ± 3.30	0.179
P*	0.928	0.016	0.058	0.122	
WC (cm)^a^					
Before	86.75 ± 7.23	92.00 ± 9.01	89.23 ± 8.37	86.14 ± 6.72	0.071
After	86.85 ± 7.37	91.09 ± 8.68	88.61 ± 8.25	85.04 ± 6.49	0.077
P*	0.755	0.008	0.039	0.017	
PA^b^	120.39 (49.50, 693.00)	153.10 (49.50, 925.00)	152.47 (49.50, 808.5)	135.12 (49.50, 694.00)	0.874

*Mg* magnesium, *BMI* body mass index, *WC* waist circumference, *PA* physical activity

*P* values based on One-Way ANOVA for continuous variables and χ2 for categorical variables

P* values based on paired-samples t test

^a^Values are Means ± SD

^b^Values are Geometric Means (Min, Max)

^c^Values are expressed as frequency (%)

**Fig. 2:**
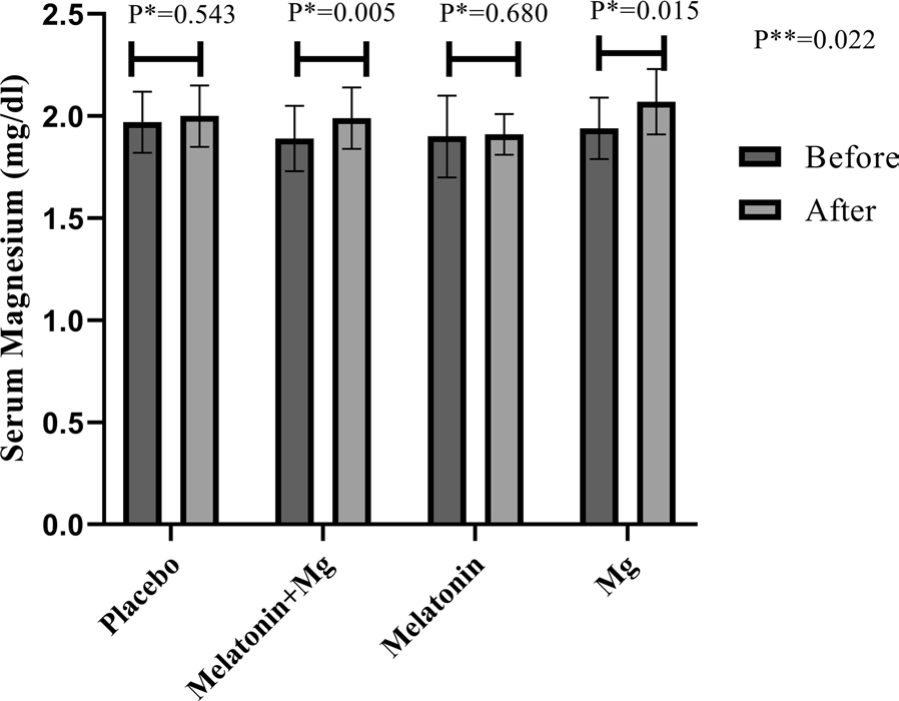
Serum magnesium levels of study participants throughout the study. P* value based on paired-samples t test. P** values based on Analysis of Covariance (ANCOVA) after adjustment of baseline values. *Mg* magnesium

As summarized in Fig. 2, there was a significant increase in serum magnesium levels in magnesium or combined magnesium plus melatonin groups (*P* < 0.05), and a greater increase was found among those who took magnesium compared with the other groups (*P* = 0.022).

Based on Table 2, there were no significant differences in the dietary intakes between the four groups before the intervention (*P* > 0.05).

**Table 2:** Dietary intakes of study participants before the intervention

Variables	The study groups				*P*
	Placebo (n = 20)	Melatonin + Mg (n = 22)	Melatonin (n = 21)	Mg (n = 21)	
Energy (Kcal)	1325.66 ± 252.63	1277.90 ± 273.18	1449.01 ± 400.07	1256.00 ± 396.07	0.259
Carbohydrate (g)	191.75 ± 47.68	176.08 ± 41.24	195.69 ± 58.17	174.78 ± 59.92	0.116
Protein (g)	48.87 ± 14.12	46.80 ± 14.77	58.02 ± 16.71	49.64 ± 17.54	0.461
Fat (g)	40.90 ± 13.82	43.76 ± 15.87	48.79 ± 15.97	40.93 ± 17.17	0.336
PUFA (g)	5.88 (1.06, 28.47)	7.59 (0.87, 24.66)	7.68 (1.92, 20.01)	6.42 (1.33, 22.70)	0.593
MUFA (g)	11.93 ± 5.11	12.75 ± 5.95	14.73 ± 5.86	11.97 ± 6.64	0.386
SFA (g)	13.71 ± 5.01	13.08 ± 5.19	16.97 ± 8.43	12.44 ± 6.77	0.120

Data are are Means ± SD and Geometric Means (Min, Max)

*P* values based on One-Way ANOVA test or Kruskal–Wallis test

*PUFA* polyunsaturated fatty acid, *MUFA* monounsaturated fatty acid, *SFA* saturated fatty acid

After two months of supplementation, the mean PSQI score decreased significantly (indicating sleep improvement) in both the magnesium-melatonin co-supplementation and melatonin groups (*P* < 0.001). When we controlled the analysis for baseline values and energy intake, patients who consumed melatonin alone or in combination with magnesium had a greater improvement in sleep quality than other categories (*P* < 0.001). The effects of magnesium, melatonin, and combined magnesium plus melatonin supplementation on the metabolic and hormonal parameters are presented in Table 3. Women who received magnesium- melatonin had significantly reduced glucose homeostasis parameters (insulin and HOMA-IR), serum cholesterol, LDL-C, and testosterone levels, as compared to the baseline values (*P* < 0.05). Although melatonin administration for eight weeks to women with PCOS decreased circulating levels of testosterone (*P* < 0.05), those who received the combination of magnesium plus melatonin experienced a greater decrease in testosterone concentrations compared with the placebo (*P* < 0.05). An increase in serum high-density lipoprotein cholesterol (HDL-C) levels was also observed following the administration of the melatonin alone or in combination with magnesium (*P* < 0.05). As well, within-group differences revealed a significant increase in FAI and circulating level of insulin in the placebo group (*P* < 0.05) (Table 3).

**Table 3:** Comparison of metabolic and hormonal parameters in study groups before and after the intervention

Variables		The study groups							P**
		Placebo (n = 20)	Melatonin + Mg (n = 22)		Melatonin (n = 21)		Mg (n = 21)		
		Mean ± SD	Mean ± SD	AMD	Mean ± SD	AMD	Mean ± SD	AMD	
^#^ PSQI	Before	5.58 (1.00, 17.00)	6.45 (3.00, 13.00)	− 0.31	5.65 (1.00, 13.00)	− 0.29	4.30 (1.00, 13.00)	− 0.08	< 0.001
	After	3.99 (1.00, 17.00) ^ab^	3.52 (1.00, 7.00) ^cd^		3.18 (1.00, 12.00) ^cd^		3.82 (1.00, 13.00) ^ab^		
	GMD	− 1.59 (− 28.49)	− 2.93 (− 45.42)		− 2.47 (− 43.71)		− 0.48 (− 11.16)		
	P*	0.17	< 0.001		< 0.001		0.155		
FBS (mg/dl)	Before	80.90 ± 5.59	88.68 ± 11.64	0.60	82.47 ± 8.09	− 0.86	82.04 ± 7.05	0.48	0.930
	After	81.35 ± 7.29	85.23 ± 9.23		81.41 ± 8.71		82.19 ± 8.04		
	MD	0.45 ± 6.56	− 3.45 ± 11.45		− 1.05 ± 9.05		0.14 ± 7.17		
	P*	0.761	0.172		0.599		0.928		
^#^Insulin (mIU/mL)	Before	6.40 (1.30, 67.90)	6.94 (1.70, 21.00)	− 0.15	7.42 (2.80, 67.90)	− 0.06	6.29 (2.30, 45.60)	− 0.07	0.169
	After	6.98 (1.50, 22.40)	5.83 (1.50, 11.00)		7.87 (2.90, 20.10)		6.74 (3.00, 15.50)		
	GMD	0.58 (9.06)	− 1.11 (− 15.99)		0.45 (6.06)		0.45 (7.15)		
	P*	0.049	0.010		0.837		0.653		
^#^HOMA-IR	Before	1.31 (0.25, 14.08)	1.50 (0.37, 5.00)	− 0.07	1.48 (0.48, 14.08)	− 0.03	1.27 (0.44, 9.23)	− 0.03	0.355
	After	1.41 (0.32, 5.83)	1.22 (0.32, 2.61)		1.57 (0.48, 4.81)		1.36 (0.58, 3.64)		
	GMD	0.10 (7.63)	− 0.28 (− 18.66)		0.09 (6.08)		0.09 (7.08)		
	P*	0.053	0.006		0.859		0.662		
^#^HOMA-B	Before	120.00 (24.48, 896.00)	108.06 (24.48, 290.77)	− 0.15	140.25 (38.67, 622.80)	− 0.01	127.70 (55.20, 864.00)	− 0.05	0.235
	After	138.04 (23.13, 979.20)	104.78 (23.13, 540.00)		167.69 (51.23, 590.40)		139.25 (54.58, 979.20)		
	GMD	18.04 (15.03)	− 3.28 (− 3.03)		27.44 (19.56)		11.55 (9.04)		
	P*	0.097	0.802		0.479		0.611		
TG (mg/dl)	Before	133.00 ± 50.05	159.59 ± 80.90	29.77	129.42 ± 73.18	11.45	143.23 ± 69.63	23.03	0.158
	After	116.66 ± 37.97	157.96 ± 67.51		126.41 ± 46.00		144.96 ± 66.86		
	MD	− 16.33 ± 44.88	4.00 ± 61.87		− 3.01 ± 55.45		1.72 ± 50.30		
	P*	0.120	0.770		0.806		0.877		
Cholesterol (mg/dl)	Before	160.90 ± 24.07	175.81 ± 34.67	− 3.26	162.85 ± 29.98	8.55	164.76 ± 28.80	3.97	0.122
	After	153.22 ± 20.29	159.73 ± 23.54		162.11 ± 24.45		160.09 ± 27.11		
	MD	− 7.67 ± 19.54	− 16.08 ± 18.40		− 0.73 ± 20.94		− 4.66 ± 20.71		
	P*	0.095	0.001		0.873		0.315		
LDL-C (mg/dl)	Before	91.56 ± 20.88	101.30 ± 28.55	− 8.72	94.38 ± 22.99	3.55	92.42 ± 29.19	− 2.53	0.070
	After	84.70 ± 18.03	82.33 ± 15.72		91.68 ± 21.65		85.99 ± 22.41		
	MD	− 6.86 ± 18.05	− 18.96 ± 22.02		− 2.70 ± 17.26		− 6.42 ± 20.62		
	P*	0.106	0.001		0.481		0.169		
HDL-C (mg/dl)	Before	42.73 ± 7.22	42.60 ± 11.47	0.98	42.58 ± 8.78	1.60	43.68 ± 8.72	0.43	0.615
	After	43.92 ± 6.87	44.79 ± 11.31		45.35 ± 9.62		45.22 ± 7.60		
	MD	1.19 ± 2.91	2.19 ± 3.56		2.76 ± 4.81		1.54 ± 4.72		
	P*	0.084	0.009		0.016		0.151		
^#^Testosterone (ng/ml)	Before	0.34 (0.10, 1.20)	0.36 (0.10, 1.20)	− 0.04	0.40 (0.10, 0.90)	− 0.02	0.28 (0.10, 0.70)	− 0.03	0.017
	After	0.31 (0.10, 0.90^)a^	0.27 (0.10, 0.90) ^d^		0.34 (0.10, 0.70)		0.26 (0.10, 0.80)		
	GMD	− 0.03 (− 8.82)	− 0.09 (− 25.00)		− 0.06 (− 15.00)		− 0.02 (− 7.14)		
	P*	0.08	< 0.001		0.03		0.713		
^#^SHBG (nmol/l)	Before	23.18 (5.20, 84.60)	21.82 (5.20, 84.60)	− 0.001	19.66 (5.60, 76.40)	− 0.006	27.69 (14.00, 72.70)	0.04	0.720
	After	20.79 (4.90, 115.00)	19.35 (5.60, 115.00)		17.66 (4.90, 58.82)		25.95 (15.30, 57.00)		
	GMD	− 2.39 (− 10.31)	− 2.47 (− 11.31)		− 2.00 (− 10.17)		− 1.74 (− 6.28)		
	P*	0.065	0.077		0.142		0.621		
^#^FAI	Before	1.49 (0.26, 12.50)	1.69 (0.32, 9.62)	− 0.10	2.05 (0.26, 12.50)	− 0.04	1.01 (0.26, 5.00)	− 0.09	0.161
	After	1.51 (0.17, 14.29)	1.40 (0.17, 5.68)		1.95 (0.26, 14.29)		1.01 (0.18, 4.73)		
	GMD	0.02 (1.34)	− 0.29 (− 17.15)		− 0.10 (− 4.87)		0 (0)		
	P*	0.011	0.112		0.648		0.985		
^#^Leptin (ng/ml)	Before	31.07 (4.60, 72.30)	35.71 (8.40, 93.40)	− 0.03	35.58 (13.60, 114.20)	− 0.02	35.50 (14.70, 78.60)	− 0.06	0.645
	After	31.46 (6.60, 84.10)	32.55 (14.10, 74.78)		32.93 (15.00, 103.00)		30.28 (8.10, 78.60)		
	GMD	0.39 (1.25)	− 3.16 (− 8.84)		− 2.65 (− 7.44)		− 5.22 (− 14.70)		
	P*	0.866	0.226		0.180		0.189		

Means ± SD are presented for normally distributed data

^#^Data are Geometric Means (Min, Max) and Difference in geometric means (percent change percent in geometric means)

P* values based on paired sample t test

P** values based on Analysis of Covariance (ANCOVA) followed by Sidak’s test after adjustment of baseline values and energy intake

AMD (Adjusted Mean Difference): Adjusted mean_Treatment_ – Adjusted mean_placebo_; MD (Mean Difference): Mean_after_ – Mean_before_

^a^*P* value less than 0.05 vs. melatonin and magnesium group

^b^*P* value less than 0.05 versus melatonin group

^c^*P* value less than 0.05 versus magnesium group

^d^*P* value less than 0.05 versus placebo group

*PSQI* Pittsburgh Sleep Quality Index, *FBS* fasting blood sugar, *HOMA-IR* homeostatic model assessment for insulin resistance, *HOMA-B* homeostasis model of assessment β cell function, *TG* triglycerides, *HDL-C* high density lipoprotein-cholesterol, *LDL-C* low density lipoprotein-cholesterol, *SHBG* sex hormone-binding globulin, *FAI* free androgen index, *GMD* geometric mean difference

## Discussion

The present four-arm, parallel, double-blind randomized controlled trial was designed to evaluate the independent and additive effects of magnesium and melatonin on metabolic profiles and levels of sex hormones in women with PCOS. We found that combination therapy with magnesium and melatonin in patients with PCOS had beneficial effects on sleep quality and total testosterone. Additionally, melatonin supplementation alone was found to be associated with a significant reduction in PSQI score in PCOS subjects.

PCOS is one of the most common metabolic disorder which is characterized by hyperinsulinemic- and hyperandrogenic-related disorders [37]. Since insulin resistance is an important etiological feature of PCOS, affected women are at higher risk of developing type 2 diabetes and related metabolic complications [38]. On the other hand, it has been reported that sleep disturbances are common in PCOS, and some form of them, like obstructive sleep apnoea, in turn, exacerbates insulin resistance [39]. In accordance with our results, there were studies that have shown treatment with exogenous melatonin had favorable effects on sleep quality, which was assessed by the PSQI, especially in the adults with metabolic disorders [40]. As melatonin is known to be an effective regulator of circadian rhythm and promotes sleep [41], beneficial effects on sleep quality are not surprising. In several studies, it has been demonstrated that urinary excretion of 6-sulfatoxymelatonin, the main excretory metabolite of melatonin, along with the melatonin levels in blood and saliva was higher in patients with PCOS than women with normal fertility and the levels of these molecules (urinary 6-sulfatoxymelatonin and serum melatonin) were significantly correlated with the severity of sleep disturbances [42]. Although the causal role of melatonin in the pathogenesis of PCOS and sleep disorders is still unclear, the production of higher amounts of melatonin in women with PCOS may be in an effort of eliminating extra free radicals as PCOS patient's high oxidative stress [43].

One of the main findings of the present study was the beneficial effects of melatonin (alone or in combination) on testosterone. In line with our finding, Jamilian and colleagues indicated that melatonin administration for three months to PCOS women significantly reduced total testosterone [44]. In another study, long-term (6 months) melatonin administration to women with PCOS had beneficial effects on menstrual irregularities and biochemical hyperandrogenism [45]. Prior studies have indicated hyperandrogenism is closely correlated with chronic inflammation and oxidative stress [46]. There is promising evidence that shows melatonin is a powerful free radical scavenger and effective endogenous antioxidant which exerts protective effects, particularly in female reproductive organs [47]. Additionally, the anti-inflammatory activity of magnesium and also its effects on improved insulin sensitivity have been previously shown [48, 49]. Although our study failed to find any significant effect of magnesium supplementation on glycemic indices (FBS, insulin, and HOMA-IR), taking magnesium plus melatonin supplements had protective effects on insulin and HOMA-IR in women with PCOS. This result agrees with some previous studies in which melatonin administration significantly improved glucose homeostasis and insulin resistance in women with PCOS [25]. It seems that melatonin through melatonin receptors 1 and 2, suppresses hepatic gluconeogenesis and improves glucose uptake by peripheral tissues [50, 51]. In other words, melatonin administration exerts antihyperglycemic effects and improves glucose hemostasis [52].

Another key finding of the present study is that combined magnesium and melatonin supplementation was more effective in improving metabolic parameters, including cholesterol, LDL-C, and HDL-C. Moreover, supplementation with melatonin alone significantly increased serum HDL-C levels in PCOS women. These findings are consistent with growing evidence from both animal and human studies [23, 25]. For instance, Shabani and colleagues have shown that melatonin supplementation to patients with PCOS significantly decreased serum LDL-cholesterol levels [25]. Another study designed by Raygan et al. demonstrated that supplementation with melatonin (10 mg/day) for 12 weeks to diabetic people with coronary artery disease had beneficial effects on HDL-C concentrations, glycemic control, and insulin sensitivity [24]. Similarly, the results of a meta-analysis study showed noticeable effects of melatonin intake on the serum triglycerides and total cholesterol levels while did not influence HDL-C and LDL-C concentrations [53]. These inconsistencies in results of these studies may be due to differences in dosage of melatonin intake, type of diseases, duration and design of the intervention, or ethnic background of the participants. Melatonin may exert protective impacts on lipid profile by increasing lecithin-cholesterol acyltransferase activity [54]. The synergistic influence of co-administration of melatonin and magnesium in this study is also supported by some trials that indicated magnesium supplementation might help improve metabolic profiles in women with PCOS [28]. In this regard, magnesium and vitamin E co-supplementation for 12 weeks to individuals with PCOS led to a significant reduction in serum triglycerides, VLDL, and total cholesterol [28]. Magnesium seems to improve lipid concentrations through enhanced lipoprotein lipase activity [55] and increased excretion of fecal fat [56]. Despite the fact that we failed to find any significant effect of melatonin and/or magnesium on serum levels of leptin, animal studies have previously shown that melatonin administration could attenuate the development of hyperinsulinemia [57]. In fact, melatonin appears to regulate leptin synthesis, and lack of melatonin or knocking out the melatonin receptors can lead to leptin resistance [58].

One of the major concerns in PCOS patients is infertility which results mainly from follicular atresia, anovulation, and hyperandrogenemia [59, 60]. Thus, with regard to beneficial effects of both melatonin and magnesium on menstrual cycle improvement, administration of the two compounds, as therapeutic agents, to manage infertile patients in whom infertility occurs due to poor oocyte quality and anovualtion may create a new ray of hope for infertile patients [59–61]. However, the exact effect of long term melatonin and magnesium therapy on menstrual cyclicity and subsequently on the management of infertility needs to be evaluated in large scale prospective randomized studies.

It is worth noting that this is the first study, to our best knowledge, that has evaluated additive effects of melatonin and magnesium on the metabolic profile of patients with PCOS. Nevertheless, several limitations need to be addressed. Relatively short duration of supplementation should be considered in the interpretation of our findings, and long-term trials among various ethnic groups are needed to provide better effects. In addition, as the current research was conducted using relatively small number of eligible participants, the generalization of our findings is partly limited. Besides, 24-h dietary recall, as a memory dependent tool, is frequently associated with underestimation and represents only recent dietary patterns rather than a long term practice,. Thus, a valid and reliable food frequency questionnaire, (if available) may be much helpful in better estimating nutritional status of the participants and its potential effects on bio metabolic parameters of the patients.

## Conclusions

Altogether, our study demonstrated that melatonin and magnesium co-supplementation, for eight weeks in women with PCOS had beneficial effects on sleep quality and total testosterone. Additionally, melatonin supplementation alone was found to be associated with a significant reduction in PSQI score. Moreover, combined magnesium and melatonin supplementation was more effective in improving metabolic parameters, including serum levels of cholesterol, LDL-C, HDL-C, insulin, and HOMA-IR. Further large-scale research with longer periods is needed to get stronger results.

## Data Availability

The data gathered and analyzed during the current study are available from the corresponding author on reasonable request.

## Abbreviations

BMI: body mass index
DHEA: dehydroepiandrosterone
FAI: free androgen index
FBS: fasting blood sugar
HDL-C: high-density lipoprotein cholesterol
HOMA-B: homeostasis model of assessment β cell function
HOMA-IR: homeostasis model of assessment-insulin resistance
IR: insulin resistance
LDL-C: low-density lipoprotein cholesterol
LH: luteinizing hormone
MetS: metabolic syndrome
PSQI: Pittsburg sleep quality index
QUICKI: quantitative insulin sensitivity check index
SHBG: sex hormone-binding globulin
TC: total cholesterol
TG: triglycerides
WC: waist circumference
